# Psychometric properties of an arabic translation of the short form of Weinstein noise sensitivity scale (NSS-SF) in a community sample of adolescents

**DOI:** 10.1186/s40359-023-01433-7

**Published:** 2023-11-08

**Authors:** Noad Maria Azzi, Vanessa Azzi, Rabih Hallit, Diana Malaeb, Mariam Dabbous, Fouad Sakr, Feten Fekih-Romdhane, Sahar Obeid, Souheil Hallit

**Affiliations:** 1 School of Medicine, Université de Strasbourg, Strasbourg, France; 2 School of Medicine and Medical Sciences, Holy Spirit University of Kaslik, P.O. Box 446, Jounieh, Lebanon; 3 Department of Infectious Disease, Bellevue Medical Center, Mansourieh, Lebanon; 4 Department of Infectious Disease, Notre Dame des Secours, University Hospital Center, Byblos, Lebanon; 5 College of Pharmacy, Gulf Medical University, Ajman, United Arab Emirates; 6 School of Pharmacy, Lebanese International University, Beirut, Lebanon; 7 École Doctorale Sciences de la Vie et de la Santé, Université Paris-Est Créteil, Institut Mondor de Recherche Biomédicale, Paris, France; 8 The Tunisian Center of Early Intervention in Psychosis, Department of psychiatry “Ibn Omrane”, Razi hospital, Manouba, 2010 Tunisia; 9 Faculty of Medicine of Tunis, Tunis El Manar University, Tunis, Tunisia; 10 Social and Education Sciences Department, School of Arts and Sciences, Lebanese American University, Jbeil, Lebanon; 11 Psychology Department, College of Humanities, Effat University, Jeddah, 21478 Saudi Arabia; 12 Applied Science Research Center, Applied Science Private University, Amman, Jordan; 13 Research Department, Psychiatric Hospital of the Cross, Jal Eddib, Lebanon

**Keywords:** Noise sensitivity, Noise annoyance, Insomnia severity, Psychometrics, Adolescents, Lebanon, Arabic

## Abstract

**Background:**

The Weinstein Noise Sensitivity Scale (NSS) is widely recognized as a prominent unidimensional self-reported questionnaire to measure noise sensitivity, which is regarded as the foremost subjective factor moderating the impact of noise on perceived levels of annoyance. In this current study, we conducted an examination of the psychometric properties of a newly translated Arabic version of the short form of the scale (NSS-SF).

**Methods:**

A sample of 527 Lebanese adolescents participated in the study, completing the NSS-SF with measures of noise annoyance and insomnia. The total sample was randomly divided into two subsamples. Exploratory-to-Confirmatory Factor Analysis (EFA-CFA) was conducted. The normed model chi-square (χ²/df), the Steiger-Lind root mean square error of approximation (RMSEA), the Tucker-Lewis Index (TLI) and the comparative fit index (CFI). Values ≤ 5 for χ²/df, and ≤ 0.08 for RMSEA, and 0.90 for CFI and TLI indicate good fit of the model to the data. Composite reliability in both subsamples was assessed using McDonald’s ω and Cronbach’s alpha.

**Results:**

EFA results on the first split-half subsample revealed that one item (item 2) was removed because of low communality (< 0.3); the other 4 items converged on one factor, which explained 67.85% of the common variance (ω = 0.84 and α = 0.84). CFA was conducted on the second half-split subsample in adults according to the model obtained on the first split-half subsample; the fit indices were acceptable as follows: χ^2^/df = 5.07/2 = 2.54, *p* < .001, RMSEA = 0.076 (90% CI 0.001, 0.160), SRMR = 0.021, CFI = 0.992, and TLI = 0.976 (ω = 0.84 and α = 0.84). Configural, metric, and scalar invariance was supported across gender in the total sample.

**Conclusion:**

Our findings substantiate that the Arabic version of the NSS-SF is a reliable, psychometrically valid instrument for assessing noise sensitivity among Arab adolescents, thereby enhancing its overall utility and applicability within Arab countries.

## Background

Noise has been widely perceived as a prototypical environment stressor exerting a significant impact on public health [1], warranting its inclusion in the highest priority category on the World Health Organization’s list of environmental stressors [2]. It has been associated with a host of adverse effects, exhibiting a range of reactions and responses that are contingent upon individual variability in the perception of the sound [3]. Early studies have documented marked associations between noise annoyance reactions and noise exposure level [4]. However, the noise exposure level may not be the sole determination of individual’s reactions, as studies have reported that the same degree of exposure to noise in two individuals may not necessarily result in the same degree of annoyance or other non-auditory health effects [5]. Instead, it has been demonstrated that various personal characteristic factors can signficantly influence individual’s reactions to noise [6, 7]. Among these factors, individual noise sensitivity has been identified as a significant contributor to the variability in reactions to noise within the same acoustical conditions [8].

Noise sensitivity has been labeled a stable, subjective attitudinal trait, while being invariant across noise exposure levels, and represents an independently contributing factor to noise annoyance reactions [9]. In the general population, the prevalence of highly noise sensitive individuals was estimated to vary between 12 and 15% [10]. In the extant literature, subjective sensitivity to noise has been extensively researched and was found to be highly correlated with non-auditory harmful effects of noise [11]: noise-induced sleep disturbances, mostly insomnia [12], as well as the development of psychotic disorders and mental disorders [11], by strongly influencing personal reactions to environmental noise. Thus, noise sensitivity may mediate or moderate the influences of noise exposure on health [13]. Consequently, the inclusion of listener characteristics in sound research becomes imperative to enable accurate distinction between effects attributed to the stimuli itself and effects influenced by individual factors. Given the recognized importance of noise sensitivity as an influential individual difference variable in the perception and assessment of noise-related outcomes, it is crucial that researchers incorporate and quantify this variable by developing specific measurement instruments and scales, before the noise-induced health outcomes can be adequately evaluated [11].

Multiple scales have been developed to assess noise sensitivity [14, 15], but the most widely adopted and extensively validated one is the Weinstein’s noise sensitivity scale (NSS) [16]. The NSS is a 21-item scale describing individual affective reactions to a wide array of everyday environmental sounds, each item being measured along a 6-point Likert scale ranging from 1 “strongly disagree” to 6 “strongly agree”. This scale has been demonstrated to have strong internal consistency (α = 0.86) as well as satisfactory psychometric properties [17] possessing strong reliability, internal consistency, factor structure, and construct validity. Moreover, while examining the intricacies of noise sensitivity and its implications, it has been found that the incorporation of demographic variables, which encompasses subjective factors such as age and gender, significantly influences noise sensitivity [18]. Nevertheless, studies have verified the property of gender invariance, a finding which added evidence of the scale’s psychometric property [19]. Since the development of Weinstein’s noise sensitivity scale, it has been widely translated and validated into different languages, including but not limited to Swedish [17], Italian [18], German [19], Japanese [20], Persian [21], Chinese [22] and Turkish [23]. However, the full version of the NSS was deemed to be excessively lengthy for effective administration in time-sensitive field settings. In fact, previous research has demonstrated that the compliance rate of participants consenting to intercept interviews decreases as the length of questionnaires increases [24]. Accordingly, the full 21-item NSS was shortened into a condensed alternative version, the Short Form of Weinstein Noise Sensitivity Scale (NSS-SF), which was developed in a US sample [24], specifically tailored to be more time-efficient and suitable for field settings, while still maintaining and upholding the psychometric properties, reliability, validity, fidelity and effective representativeness of the original scale [25]. The NSS-SF has been validated in Bulgarian [25] and Chinese [19]. Studies have consistently demonstrated a significant correlation between noise annoyance and noise sensitivity identified by the NSS-SF [24], which added an additional level of validity to the scale. Additionally, results have shown that the NSS-SF was deemed psychometrically similar to the longer scale. In fact, in the NSS-SF, CFA fit indices produced high loadings and internal consistency measures [24]. Overall, studies demonstrated the validation of the scale on different levels; showing adequate temporal consistency, linguistical validity, as well as nomological, convergent, and discriminant validity [25].

## The present study

The aim of the present study was to develop an Arabic version of the NSS-SF and assess its psychometric properties within a community sample of adolescents, thereby enhancing its overall utility and applicability within Arab countries. Furthermore, in light of existing literature highlighting significant associations between noise sensitivity and individual or internal factors, an important objective of the present study was to assess the scale’s dimensionality and reliability by investigating the influence of age and sex on noise sensitivity [18, 19, 26]. To ensure the validity and robustness of cross-group comparisons, measurement invariance (MI) tests will be conducted to identify and mitigate any potential measurement artifacts. Moreover, the study aimed to examine the nomological validity and equivalence of the NSS-SF by analyzing its association with measures of insomnia and noise annoyance. These measures were included based on previous literature demonstrating an association between noise sensitivity and sleep disturbances, including insomnia [27–29], as well as between noise sensitivity and noise annoyance [30–32]. We hypothesize that the Arabic version will have a unidimensional structure, have similar psychometric properties in terms of internal consistency and will have invariant measurement between genders.

## Methods

### Participants and procedures

A total of 527 adolescents completed the survey (mean age: 15.73 ± 1.81; 56% females). A convenient sampling method (snowball technique) was used to collect data during April-May 2023. After completing a training with the research team, eight university students were asked to collect data via a Google Form link; they were asked to forward the link to people they know, who in turn were asked to forward the link to other family members and friends. Inclusion criteria for participation included being of a resident and citizen of Lebanon and aged between 12 and 18 years. Excluded were those who refused to fill out the questionnaire, not being residents or citizens of Lebanon and those aged under or above 12–18 years. Internet protocol (IP) addresses were examined to ensure that no participant took the survey more than once. Informed consent was obtain from parents as well. Participants were asked in the introductory paragraph to take their parents’ consent before filling the survey. After providing digital informed consent, participants were asked to complete the instruments described above, which were presented in a pre-randomised order to control for order effects. The survey was anonymous and participants completed the survey voluntarily and without remuneration.

### Translation procedure

The forward-backward translation approach was used for the NSS-SF. The English version was translated to Arabic by a Lebanese translator who was completely unrelated to the study. Afterwards, a Lebanese psychologist with a full working proficiency in English, translated the Arabic version back to English. The translation team ensured that any literal and/or specific translation was balanced. The initial and translated English versions were compared to detect/eliminate any inconsistencies and guarantee the accuracy of the translation by a committee of experts composed of the research team, one psychologist, one psychiatrist and the two translator. An adaptation of the measure to the Arab context was performed, and sought to determine any misunderstanding of the items wording as well as the ease of items interpretation; therefore, ensure the conceptual equivalence of the original and Arabic scales in both contexts [33]. After the translation and adaptation of the scale, a pilot study was done on 30 participants to ensure all questions were well understood; no changes were applied after the pilot study.

### Measures

#### Demographics

Participants were asked to provide their demographic details consisting of age and gender.

#### The short form of weinstein noise sensitivity scale (NSS-SF)

The scale is composed of five items scored on a 6 point Likert scale (1 = strongly disagree to 6 = strongly agree). Higher agreement on a statement indicates higher noise sensitivity of the respondent [16, 34].

#### Noise annoyance

Participants were asked to answer one question “Does noise at home or work annoy you?”, with five ordered levels of response from ‘never’ to ‘always’ [13].

#### Insomnia severity index

Validated in Arabic [35], this scale is composed of 7 items rated on a four-point Likert scale. Higher scores indicate more severe insomnia. (ω = 0.68 and α = 0.59)

### Analytic strategy

#### Data analysis

There were no missing responses in the dataset. In order to study the factor structure of the scale, the total was divided into 2 subsamples; no difference was found between the two subsamples in terms of age (15.79 ± 1.87 vs. 15.68 ± 1.74; *t* = 0.710; df = 525; *p* = .478) and gender (χ^2^ = 0.001; df = 1; *p* = .981). The first subsample was used to conduct the Exploratory Factor Analysis (EFA). The Kaiser-Meyer-Olkin (KMO) measure of sampling adequacy and Bartlett’s test of sphericity ensured the adequacy of our sample [36]. As the scales have multiple response alternatives, the EFA was carried out using the Pearson correlation matrix and Principal Component Analysis as the estimation method. The dimensionality of the instrument was determined through the optimal implementation of Parallel Analysis [37]. We retained factor loadings greater than 0.40 [38].

#### Confirmatory factor analysis

We used data from the second split-half sample to conduct a CFA using the SPSS AMOS v.29 software. Our intention was to test the model obtained from the EFA results on the first split-half subsample. The normed model chi-square (χ²/df), the Steiger-Lind root mean square error of approximation (RMSEA), the Tucker-Lewis Index (TLI) and the comparative fit index (CFI). Values ≤ 5 for χ²/df, and ≤ 0.08 for RMSEA, and 0.90 for CFI and TLI indicate good fit of the model to the data [39]. The absence of multicollinearity was verified through tolerance values > 0.2 and variance inflation factor (VIF) values < 5. Multivariate normality was verified using the Bollen-Stine bootstrap *p* value = 0.255. Evidence of convergent validity was assessed in this subsample using the average variance extracted (AVE) values of ≥ 0.50 considered adequate [40].

#### Gender invariance

To examine gender invariance of NS scores, we conducted multi-group CFA [41] using the total sample. Measurement invariance was assessed at the configural, metric, and scalar levels [42]. Measurement invariance was determined if ΔCFI ≤ 0.010 and ΔRMSEA ≤ 0.015 or ΔSRMR ≤ 0.010 [41]. If invariant, we aimed to check for a difference in NS scores in terms of gender using the Student *t*-test.

### Further analyses

Composite reliability in both subsamples was assessed using McDonald’s ω and Cronbach’s alpha [43], with values greater than 0.70 reflecting adequate composite reliability. The total NS scores followed a normal distribution, with skewness and kurtosis values varying between − 1 and + 1 [44]. To assess convergent and concurrent validity, we examined bivariate correlations between NS scores and the other scales included in the survey using the Pearson test. Based on Cohen [45], values ≤ 0.10 were considered weak, ~ 0.30 were considered moderate, and ~ 0.50 were considered strong correlations.

## Results

The description of the items of the noise sensitivity scale is shown in Table 1.

**Table 1:** Description of the items of the Short Form of Weinstein Noise Sensitivity Scale (NSS-SF).

Item number	Mean	SD	Skewness	Kurtosis
1	4.45	1.28	− 0.61	− 0.22
2	3.71	1.31	− 0.35	− 0.46
3	4.43	1.33	− 0.73	0.02
4	4.61	1.28	− 0.79	0.09
5	4.27	1.34	− 0.59	− 0.23

### Exploratory factor analysis on the total sample

KMO = 0.784 and Bartlett’s statistic χ²(6) = 426.4, p < .001 ensured the adequacy of the model. The parallel analysis advised one dimension. The results of the EFA on the first split-half subsample revealed that one item (item 2) was removed because of low communality (< 0.3); the other 4 items converged on one factor, which explained 67.85% of the common variance (ω = 0.84 and α = 0.84).

### Confirmatory factor analysis of different models

A CFA was conducted on the second half-split subsample in adults according to the model obtained on the first split-half subsample; the fit indices were acceptable as follows: χ^2^/df = 5.07/2 = 2.54, *p* < .001, RMSEA = 0.076 (90% CI 0.001, 0.160), SRMR = 0.021, CFI = 0.992, and TLI = 0.976. The standardised estimates of factor loadings were all adequate (Table 2). The convergent validity for this model was adequate, as AVE = 0.74 (ω = 0.84 and α = 0.84).

**Table 2:** Items of the NSS-SF in English and Factor Loadings Derived from the Exploratory Factor Analyses (EFA) in the First Split-Half Subsample, and Standardised Estimates of Factor Loadings from the Confirmatory Factor Analysis (CFA) in the Second Split-Half Subsample

Item	EFA	CFA		
		Total	Males	Females
(1) I get annoyed when my neighbors are noisy	0.74	0.65	0.56	0.66
(2) I find it hard to relax in a place that’s noisy.	0.87	0.81	0.80	0.85
(3) I get mad at people who make noise that keeps me from falling asleep or getting work done.	0.84	0.76	0.77	0.78
(4) I am sensitive to noise.	0.84	0.74	0.75	0.76

### Gender invariance

Indices in Table 3 indicate that configural, metric, and scalar invariance was supported across gender in the total sample. No significant difference was found between females (*M* = 10.27, *SD* = 4.52) in terms of NSS-SF scores compared to males (*M* = 10.22, *SD* = 3.93), *t*(525) = − 0.135, *p* = .893.

**Table 3:** Measurement invariance across gender

Model	χ²	*df*	CFI	RMSEA	SRMR	Model Comparison	Δχ²	ΔCFI	ΔRMSEA	ΔSRMR	Δ*df*	*p*
Configural	8.31	4	0.995	0.045	0.010							
Metric	10.89	7	0.995	0.033	0.020	Configural vs. metric	2.58	< 0.001	0.012	0.010	3	0.461
Scalar	20.42	10	0.987	0.045	0.020	Metric vs. scalar	9.53	0.008	0.012	< 0.001	3	0.023

Note. CFI = Comparative fit index; RMSEA = Steiger-Lind root mean square error of approximation; SRMR = Standardised root mean square residual

### Convergent and concurrent validity

Higher NSS-SF scores were significantly and moderately correlated with more noise annoyance (*r* = .32, *p* < .001) and weakly with more insomnia severity (*r* = .17, *p* < .001).

## Discussion

The current study yielded significant findings through the examination and validation of the Arabic version of the NSS-SF. Our results supported that the Arabic version of the scale is a reliable psychometric tool, which can be used in assessing an adolescent’s noise sensitivity and its relation to noise annoyance as well as insomnia. Indeed, this Arabic version presented a convenient assessment of noise sensitivity particularly in adolescents, which constitute one of the most vulnerable age groups to adverse noise effects [46]. Our results demonstrated the validation of the scale on different levels; owing to its satisfactory temporal consistency and linguistical validity; as well as its adequate factorial, convergent and concurrent validity. Additionally, our results are consistent with prior research, indicating that there were no significant differences across gender [19]. In terms of the factorial validity of the Arabic version, our findings align with with previous studies demonstrating the unidimensionality of the scale [22, 34]. In fact, the NSS-SF has been conclusively validated to demonstrate a satisfactory structure, a solid internal consistency, and adequate convergent validity [24, 25]. The unidimensional model was found to be adequately fitting in the present study when assessed through both exploratory factor analysis (EFA) and confirmatory factor analysis (CFA). Notably, the development of the NSS-SF initially did not require any mechanical modifications, emphasizing the scale’s sufficiency in assessing the underlying construct within a cross-cultural setting [24].

The findings of the present study further revealed that the Arabic NSS-SF exhibited adequate patterns of construct validity. Tests of convergent validity showed relationships that were consistent in terms of direction and magnitude to previous studies between noise sensitivity and both noise annoyance [30–32] and insomnia [27–29]. Noise sensitivity has been found to be a stable positive predictor of noise annoyance [3, 13, 47] reflecting a greater disposition to negative affectivity and heightened vulnerability to prototypical stressors, with a decreased rate of adaptation to noises. Additionally, previous findings have demonstrated a significant positive association between increased noise sensitivity and disturbed sleep, such as insomnia [27]; thereby compromising daytime functioning. The prevalence of insomnia was notably high among children and adolescents, particularly within the Lebanese population, with a substantial overall proportion of 19% [48], thereby emphasizing the importance of developing targeted interventions that effectively address both sleep disturbances and sensitivity to noise within prevention and intervention strategies among adolescents.

Despite the wide implementation and translation of the short form of NSS-SF into different languages [19, 25, 49], a notable limitation exists as the scale lacks a field-adapted Arabic version with valid psychometric soundness, impeding its practical implementation and hindering its effective integration. Accordingly, this absence poses a significant challenge to the rigorous assessment of noise sensitivity within the Arab population. Noise pollution has emerged as a significant and critical concern in Arab countries, particularly in recent years [50–52]. Nevertheless, a bibliometric analysis [53] highlights a notable lack of consideration given to the public health implications of environmental noise with only a few studies reporting noise annoyance and sleep disturbances in Arab countries. The concerning disregard for the public health effects stemming from noise pollution in a country facing an alarming upsurge in its incidence [54] demands prompt action and thorough research. It is imperative to conduct in-depth investigations focused on assessing noise sensitivity in order to effectively raise awareness and accurately evaluate the associated implications. Moreover, the inclusion of adolescents in the study, being one of the most vulnerable age groups to adverse noise effects, holds significant relevance [46] and enhances the applicability and generalizability of the NSS-SF, providing valuable insights into the evaluation of its psychometric properties and its assessment of noise-related health impacts.

### Limitations

The present study was subject to several limitations, warranting attention in future research endeavors. Firstly, a significant limitation of this study pertains to the method of recruitment, the sample being confined solely to Lebanese individuals, which may have impeded the representativeness of the sample in relation to the wider Arab population. This constraint should be taken into consideration when interpreting and extrapolating the study’s outcomes to other Arab communities. Prospective research endeavors should strive to adopt more diverse and representative sampling methods, thereby bolstering the external validity as well as the generalizability of the outcomes to a broader spectrum of the Arab population. Moreover, it is important to consider the potential value in assessing the generalizability and replicability of the current findings within older age cohorts. It is worth noting that Senese et al. (34) reported contrasting findings, stating that females exhibited higher levels of noise sensitivity compared to males, and individuals older than 45 years demonstrated greater noise sensitivity compared to younger individuals. Therefore, conducting an investigation into the replicability of the current study’s findings within different age groups, particularly among older adults in Lebanon and in other Arab countries would yield valuable insights and enable the assessment of discriminant validity within these specific age groups. Finally, the robust unidimensional structure of the Weinstein’s NSS has been demonstrated to persist consistently across diverse linguistic and cultural contexts [22, 34], rendering it suitable for cross-national score comparisons. Nevertheless, the lack of sufficient validation of the short version of the scale in different languages restricts our ability to compare our validation results with other cross-cultural validations of the scale. Consequently, the generalizability of the findings regarding the validation of the NSS-SF across various cultural and linguistic groups remains limited, underscoring the need for further research and validation endeavors to enhance its cross-cultural applicability.

## Conclusion

In conclusion, the findings of this present study provide solid evidence supporting the validation of the psychometric properties of the Arabic translation of the NSS-SF among a sample of adolescents. This validation process of the scale conducted in the Arabic language would provide valuable insights by eventually enabling the exploration of intricate correlations between noise sensitivity and specific health outcomes, thus contributing to a more comprehensive understanding of the public health implications of noise pollution, particularly within Arab countries.

## Data Availability

The datasets generated and/or analyzed during the current study are not publicly available due to the restrictions from the ethics committee but are available from the corresponding author on a reasonable request.
